# Reusable Mg supported IRMOF-3 as a heterogeneous nanocatalyst for chemoselective reduction of biomass-based aldehydes and ketones to alcohols under mild conditions

**DOI:** 10.1098/rsos.242257

**Published:** 2025-08-06

**Authors:** Fatemeh Moghimpour Bijani, Ali Reza Sardarian

**Affiliations:** ^1^ Department of Chemistry, Shiraz University, Shiraz, Iran

**Keywords:** biomass-derived carbonyl compounds, sustainable chemistry, chemoselective reduction, heterogeneous magnesium nanocatalyst, IRMOF-3, Di(2-pyridyl) ketone

## Abstract

A novel, durable heterogeneous magnesium-supported catalyst, IRMOF-3@Di(2-pyridyl) ketone@Mg(II) (Di(2-pyridyl) ketone: Di-2pyk), was effectively synthesized using a convenient approach and fully characterized utilizing chemo-physical analysis techniques such as powder X-ray diffraction analysis, attenuated total reflection infrared spectroscopy, field emission scanning electron microscopy with energy-dispersive spectroscopy, transmission electron microscopy, CHN analysis, inductively coupled plasma optical emission spectroscopy, thermogravimetric analysis and Brunauer–Emmett−Teller measurements. This metal–organic framework-based nanocatalyst displayed excellent catalytic behaviour in chemoselective hydrogenation of five biomass-based carbonyl compounds of furfural, cinnamaldehyde, levulinic acid, vanillin and citral in addition to a variety of aliphatic, aromatic, and α,β-unsaturated aldehydes and ketones at room temperature in water in the presence of sodium borohydride as a mild and safe hydrogen source reagent. The leaching and reusability experiments of this catalyst exhibited stability and reactivity after four runs.

## 1. Introduction

The topics of sustainable chemistry and green chemistry have garnered growing attention in light of the environmental and contaminated worldwide energy crisis. Metal complexes of the earth-abundant group 2 present environmentally sustainable and cost-effective options compared to catalysts made of expensive transition metals for various synthetic processes [1–5].

Magnesium, being classified as an alkaline-earth metal with abundance, has gained growing interest as a catalyst with environmentally benign properties [6] in a variety of organic reactions, including Grignard reactions [7], enantioselective additions [8], reductions [9], hydroborations [10] and asymmetric cyclization [11]. Its low toxicity, cost-effectiveness and ability to facilitate various transformations make it an attractive alternative to traditional transition metal catalysts. The distinctive electronic and structural characteristics of magnesium enable it to trigger reactions in manners that deviate markedly from conventional catalysts [12].

Heterogeneous catalysts offer considerable benefits, notably their recyclability resulting from their distinct phase separation from reactants, thereby enabling repeated utilization. Their solid-state characteristics permit easy separation from a reaction mixture, thereby mitigating product contamination. Moreover, the active sites of these catalysts can be regulated, thereby improving catalytic efficiency and selectivity [13,14].

Metal–organic frameworks (MOFs) represent a category of porous substances [15] consisting of clusters of metal ions that are connected to organic ligands [16], resulting in structures of one, two, or three dimensions [17]. IRMOF-3, belonging to the isoreticular MOF (IRMOF) series, has attracted considerable interest owing to its prospective utility in catalysis [18], especially in the realm of organic transformations. Characterized by its robust and highly tunable porous structure, IRMOF-3 is composed of zinc oxide clusters (Zn_4_O) interconnected by 2-aminoterephthalate ligands that offer a versatile platform for a multitude of chemical transformations such as Aldol reactions [19], Knovenagel condensation [20], Michael addition [21] and epoxide ring-opening reactions [22]. As a heterogeneous material, the capability of enabling reactions at mild conditions, in addition to its robustness and recyclability, makes it a potentially valuable material for use in various industrial settings. The existence of amino groups within the organic linkers facilitates additional functionalization, thereby enhancing the catalytic performance of IRMOF-3.

Recent research has demonstrated that subsequent post-synthetic modifications can introduce various functional groups, thereby tailoring the framework for specific catalytic purposes [23].

Reduction of carbonyl compounds is considered a fundamental reaction within the field of organic chemistry [24] with diverse applications in pharmaceuticals [25], agrochemicals [26] and materials science [27]. Recent developments in the techniques and catalysts utilized in the reduction of carbonyl compounds are discussed, with a focus on novel strategies that present enhanced effectiveness, selectivity and environmental friendliness. The ongoing investigation of reduction of carbonyl compounds remains a dynamic field of study with notable progress in catalytic [28], transfer hydrogenation [29], organocatalytic [30], electrochemical [31], photocatalytic [32] and biocatalytic [33] methods.

Catalytic hydrogenation continues to be a fundamental technique utilized in the process of reducing carbonyl compounds [34]. Traditional catalysts, such as Raney nickel and platinum-based systems [35,36], have been widely used. Recent investigations, however, have been focused on the enhancement of catalysts that are both more effective and selective. For instance, [37] reported a new class of bimetallic catalysts composed of palladium and copper in the hydrogenation of several ketones and aldehydes under mild conditions. Transfer hydrogenation, utilizing hydrogen donors like alcohols [38], formic acid [39] or NaBH_4_ [40] instead of molecular hydrogen, has attracted considerable interest owing to its operational convenience and safety.

The catalytic reduction of aldehydes and ketones can face challenges when nitro, ester, alkene, nitryl and amide functional groups are present, as they may lead to over-reduction or undesired side reactions [41]. Sodium borohydride (NaBH_4_) is considered a relatively safe and environmentally friendly, versatile and extensively utilized reducing agent within the field of organic chemistry. Due to its gentle characteristics, in the presence of an effective catalyst, it can be employed for selective reductions while other reducible functional groups are present [42,43].

The reduction of carbonyl compounds originating from biomass, such as levulinic acid [44,45], furfural [46–50], vanillin [51] and cinnamaldehyde [1,52], has become notably significant in recent years, due to the increasing focus on sustainable and renewable chemical procedures. The selective reduction of these carbonyl compounds, by employing gentle and effective reducing agents such as NaBH_4_, enables the transformation of biomass into biofuels, bioplastics and fine chemicals [53], thus playing a role in promoting the circular economy. Recent research has brought attention to the efficacy of NaBH_4_ in the transformation of biomass-derived levulinic acid and furfural into bio-based alcohols, which play a critical role as intermediates in the synthesis of polymers and pharmaceuticals [54,55].

In this project, to find an effective, eco-friendly and novel nanocatalyst using IRMOF-3 (**1**) as an efficient support with porous property, we design IRMOF-3@Di-2pyk@Mg(II) (**3**) nanocatalyst to be prepared for the green chemoselective reduction of aldehydes and ketones including biomass-based carbonyl compounds to the corresponding alcohols in the presence of sodium borohydride as mild hydrogen source under mild conditions in water instead of sing toxic and expensive organic solvents [56].

## 2. Material and methods

### 2.1. Experimental

#### 2.1.1. Preparation of IRMOF-3 (1)

**Scheme 1.**
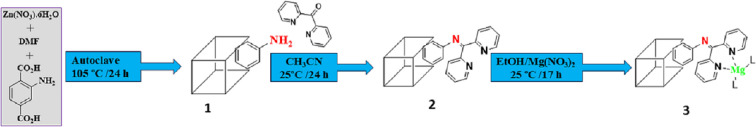
Preparation process of IRMOF-3@Di-2pyk@Mg(II) catalyst (**3**).

IRMOF-3 was synthesized following the literature procedure, with minor modifications (Scheme 1) [57]. For the typical preparation of the catalyst, Zn(NO_3_)_2_.6H_2_O (12.5 mmol) and 2-aminoterphthalic acid (H_2_ATA) (4.1 mmol) were dissolved in 50 ml anhydrous DMF and stirred for 1 h at room temperature. After that, the mixture was placed into a sealed Teflon-lined autoclave and maintained at 105°C for 24 h. The obtained slightly brown crystals were gathered by centrifugation and washed three times with EtOH and DMF. For removal of DMF from the pores, the brown crystals were immersed two times into CH_2_Cl_2_ for 24 h and subsequently dried under vacuum conditions at 150°C for 24 h.

#### 2.1.2. Preparation of IRMOF-3@Di-2pyk (2)

To synthesize IRMOF-3@Di(2-pyridyl) ketone (Scheme 1), 0.118 g of di(2-pyridyl) ketone (Di-2pyk) was dissolved in 20 ml of acetonitrile in a flask. Subsequently, 0.340 g of IRMOF-3 was added to the obtained solution. The resulting mixture was continuously stirred at room temperature for 24 h. The product with a cream colour was centrifuged and washed with a mixture of acetonitrile and ethanol (2 × 2 ml) and dried under vacuum conditions at 80°C for 12 h. Elemental analysis was subsequently carried out on the solid substance; found: C 43.63, H 4.04, N 5.52%.

#### 2.1.3. Preparation of IRMOF-3@Di-2pyk@Mg (II) (3)

To prepare IRMOF-3@Di-2pyk@Mg(II) (Scheme 1), 0.130 g of the prepared IRMOF-3@Di-2pyk was added to a flask containing 15 ml of EtOH, followed by the addition of 0.010 g of Mg(NO_3_)_2_.6H_2_O. The resulting slurry was mixed at room temperature for 17 h. Afterward, the obtained solid was centrifuged, washed carefully with ethanol (2 ×4 ml), and allowed to dry under vacuum conditions. Based on inductively coupled plasma atomic emission spectroscopy (ICP) analysis, the catalyst contained 4.97 wt% of Mg, indicating a 98% incorporation rate. To further investigate the final catalyst, elemental analysis was conducted and found: C 37.4, H 2.9, N 4.25%.

#### 2.1.4. General procedure for the catalytic reduction of carbonyl compounds to alcohols

The process of catalytic reduction of aldehydes and ketones was conducted at 25°C in a round-bottom flask for a range of 10−35 min. The system contained 2 ml H_2_O, 1 mmol aldehyde or ketone, and 2 mg IRMOF-3@Di-2pyk@Mg(II) (**3**) catalyst. At the end, 0.5 mmol NaBH_4_ was added to the mixture. The progress of the reaction was monitored using thin-layer chromatography (TLC). After adding 5 ml 0.1 M HCl solution, centrifugation was used to separate the catalyst from the reaction mixture. Ethanol (2 × 5 ml) was then used to wash the catalyst and dried under vacuum conditions at 85°C. Following purification, the prepared alcohols were characterized by ^1^H and ^13^C NMR spectra.

#### 2.1.5. Techniques for characterization

All the materials were obtained from Merck and utilized with no further purification. Powder X-ray diffraction (PXRD) analysis was carried out on a Bruker D8 ADVANCE diffractometer using Cu-Kα (λ = 1.5418 Å) radiation. Attenuated total reflection infrared (ATR-IR) and Fourier transform infrared (FTIR) spectroscopy were utilized to examine the functional groups present in both the catalyst (Bruker, ATR-IR) and the reaction products (Shimadzu FTIR-8400). Elemental and morphological analyses were conducted using energy-dispersive X-ray spectroscopy (EDS) elemental mapping and field emission scanning electron microscopy (FESEM). These analyses were performed with a Zeiss device. The images related to the size distribution of IRMOF-3@Di-2pyk@Mg(II) (**3**) were acquired using transmission electron microscopy (TEM) with an EM 208S, tungsten filament, and a 100 kV accelerating voltage. The Mg loading on the support substrate was quantified utilizing inductively coupled plasma spectroscopy (ICP) with an ICP analyser (Varian, Vista-pro). The analysis of nitrogen adsorption and desorption isotherms was performed at 77 K using a BELSORP-mini II surface area and pore size distribution analyser. Thermogravimetric analysis (TGA) was conducted using a Mettler Toledo thermal analyser, with a temperature increase of 10°C min^−1^ under a nitrogen atmosphere. The organic products were analysed using proton (^1^H) NMR and carbon (^13^C) NMR spectroscopy with a Bruker AVANCE DPX 400 MHz model.

#### 2.1.6. The catalyst characterization

As displayed in Scheme 1, IRMOF-3@Di-2pyk@Mg(II) (**3**) heterogeneous nanocomposite was synthesized through three steps. Initially, IRMOF-3 (**1**) was produced on the basis of the literature with minor revisions. In the second step, Di-2pyk was grafted on the surface of **1** using the imine reaction formation in acetonitrile at room temperature. In the end, the magnesium complex of IRMOF-3@Di-2pyk (**2**) was formed by addition of Mg(NO_3_)_2_ to a mixture of **2** in ethanol at room temperature.

**Figure 1.**
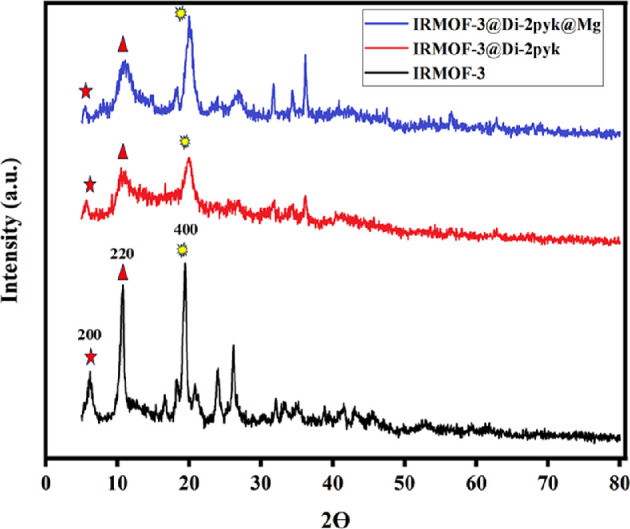
X-ray diffraction (XRD) patterns of IRMOF-3 (**1**), IRMOF-3@Di-2pyk (**2**) and fresh IRMOF-3@Di-2pyk@Mg(II) (**3**).

The crystallinity of **1**, **2** and **3** was confirmed through X-ray diffraction (XRD) analysis, as presented in figure 1. The presence of peaks at 2*θ* angles of 5.4°, 9.6° and 14.1° validates the effective formation of the three-dimensional crystal structure of **1** [58]. The catalyst **3** showed diffraction peaks similar to **1**, along with several additional peaks associated with the Di-2pyk and Mg(II). Based on the XRD pattern of the reused **3**, it appears that the overall crystal structure seems to be sustained even after four cycles (electronic supplementary material, figure S1).

**Figure 2.**
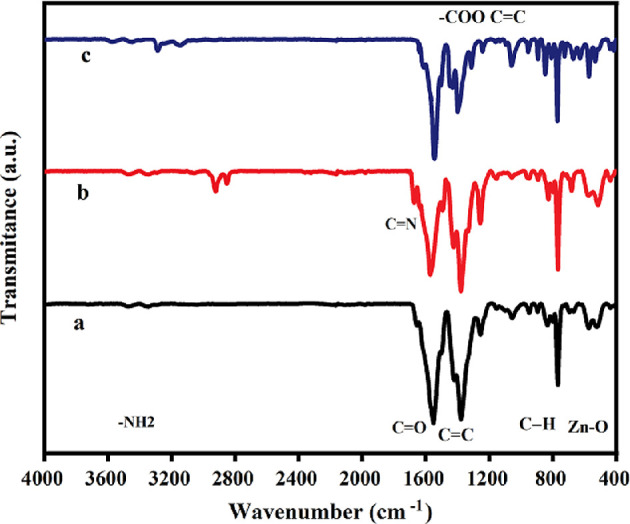
ATR-IR plots of IRMOF-3 (**1**) (a), IRMOF-3@Di-2pyk (**2**) (b) and IRMOF-3@Di-2pyk@Mg(II) (**3**) (c).

To examine the existence of organic functional groups, the ATR-IR spectra of **1**, **2** and **3** are illustrated in figure 2. The peaks related to stretching vibration, around 3480 and 3310 cm^−1^, correspond to the −NH_2_ band of free primary amine groups [59]. The peaks observed at 1657 and 1384 cm^−1^ correspond to the O–C=O asymmetric stretching vibration of carboxylic groups and C=C within the benzene ring [60]. The prominent absorption peak observed near 1574 cm^−1^ provides compelling evidence supporting the existence of the –COO symmetrical stretching vibration [57]. The spectrum of **1** exhibits peaks at 1498 and 1427 cm^−1^, signifying the stretching vibrations associated with the C=C bonds present in the benzene ring. The peak observed at 1260 cm^−1^ is designated to represent the stretching mode of the C–N bonds [59]. The absorption peaks observed at 1105 and 835 cm^−1^ are attributed to the bending vibrations of aromatic C−H bonds [60]. The presence of an absorption peak at approximately 526 cm^−1^ verifies the existence of the Zn–O bond within the Zn_4_O clusters of **1** [61]. Observation of the new peak at 1651 cm^−1^ in the infrared spectrum of **2** signifies the existence of C=N bonds and the effective modification of the IRMOF-3 framework. In the infrared spectrum of **3**, it was observed that the band associated with the C=N bond shows a downward shift to 1620 cm^−1^, indicating the interaction between the imine group and magnesium [62].

**Figure 3.**
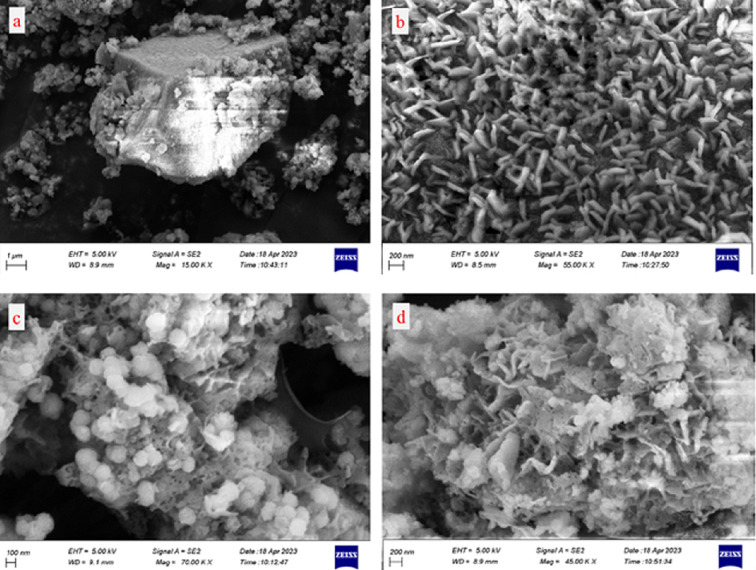
FESEM images of IRMOF-3 (**1**) (a,b), IRMOF-3@Di-2pyk (**2**) (c), and IRMOF-3@Di-2pyk@Mg(II) (**3**) (d).

**Figure 4.**
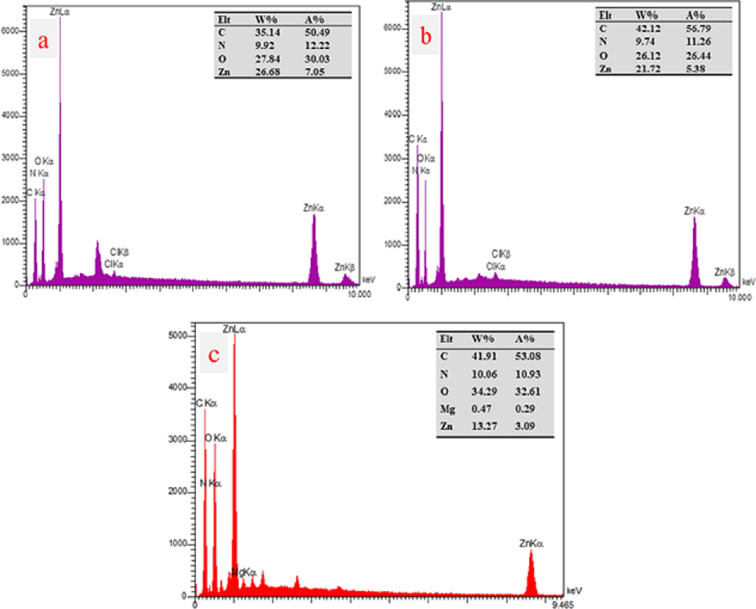
EDS spectra of IRMOF-3 (**1**) (a), IRMOF-3@Di-2pyk (**2**) (b), and IRMOF-3@Di-2pyk@Mg(II) (**3**) (c).

**Figure 5.**
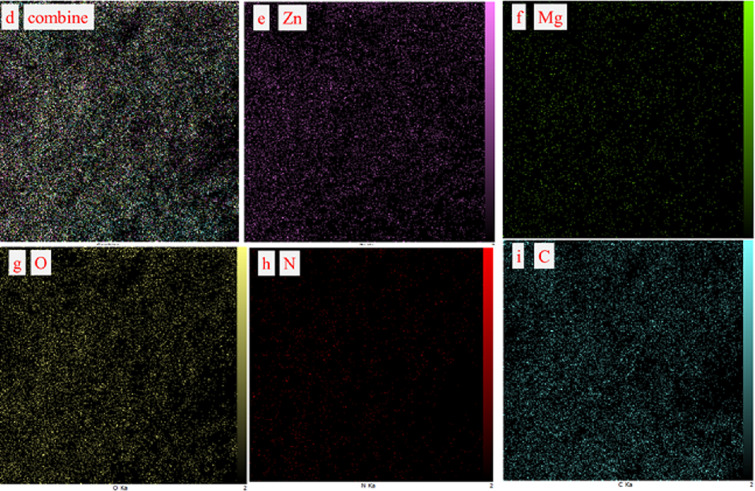
Elemental mapping of (d) combine, (e) Zn, (f) Mg, (g) O, (h) N and (i) C of IRMOF-3@Di-2pyk@Mg(II) (**3**).

The prepared samples were examined for their morphological properties utilizing FESEM. IRMOF-3 (**1**) consists of nanoparticles characterized by a uniform and smooth surface, displaying a tightly regulated distribution of crystal sizes displaying well-shaped cubic crystals (figure 3a,b). The surface morphologies of **2** and the fresh nanocatalyst are shown in (figure 3c,d). Because of the presence of Di-2pyk supported on the surface-modified framework and the interactions among neighbouring particles, the particle size of **2** and **3** was slightly larger compared to that of **1**. The EDS spectrum proved the presence of carbon (C), oxygen (O), nitrogen (N), zinc (Zn) and magnesium (Mg) elements in **1**, **2** and **3** (figure 4a–c). Additionally, elemental mapping clearly demonstrated the uniform distribution of Di-2pyk groups and magnesium nanoparticles across the surfaces of **3** without aggregation or phase separation (figure 5d–i).

**Figure 6.**
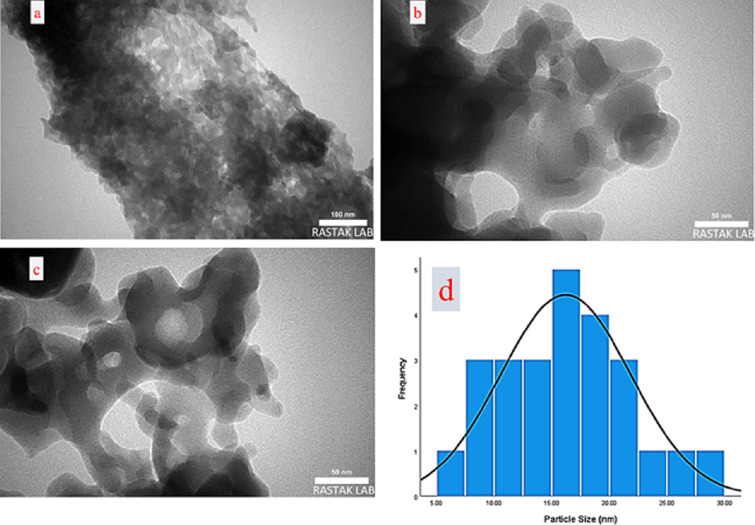
TEM images of IRMOF-3@Di-2pyk@Mg(II) (**3**) (a–c). Particle size distribution of the catalyst (d).

In order to have knowledge of the more specific internal structure and particle size of the catalyst, TEM analysis was conducted. The porous structure of **3** is distinctly visible in the TEM images (figure 6a–c). According to the TEM images, the average diameter of the nanoparticles is about 16 nm (figure 6d). The acceptable and well-arranged mode of the particles indicates the desirable monodispersities of the catalyst surface.

**Figure 7.**
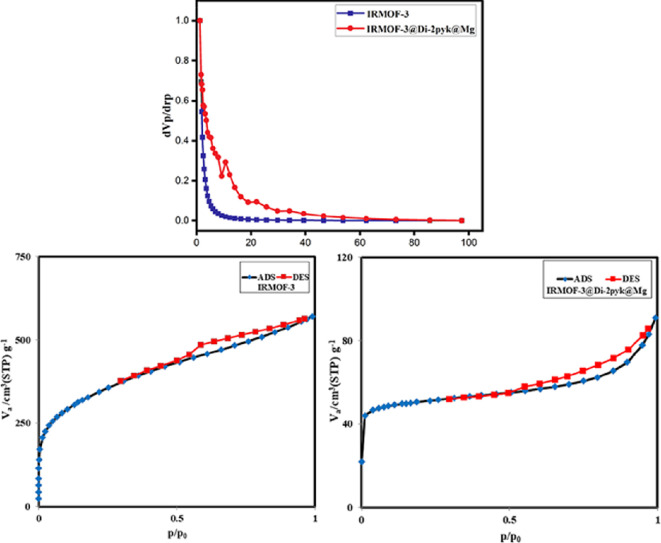
N_2_ adsorption–desorption isotherms and BJH pore-size distribution curves of IRMOF-3 (**1**) and IRMOF-3@Di-2pyk@Mg(II) (**3**).

The porosity and specific surface area of **1** and **3** were determined using the Brunauer–Emmett−Teller (BET) and the Barrett–Joyner–Halenda (BJH) methods (table 1). Figure 7 exhibits the pore size distribution curves and N_2_ adsorption–desorption isotherms for both **1** and **3**. The nitrogen sorption isotherm of **3** exhibited Type II behaviour, indicative of a partially filled microporous material with significant multilayer adsorption. A decrease in BET surface area (approx. 824.6 m² g^−1^ to approx. 238.9 m² g^−1^) and pore volume confirmed the successful grafting of di(2-pyridyl) ketone and Mg(II) ions, partially occupying internal pores. BJH pore size analysis revealed a broad micropore distribution without distinct mesoporosity, consistent with the structural integrity of the parent MOF. The presence of a minor hysteresis loop suggested slight framework flexibility upon gas adsorption. These features confirm that the functionalized catalyst maintains high surface area and accessible microporosity, suitable for efficient heterogeneous catalysis.

**Table 1.** The surface properties of IRMOF-3 (**1**) and IRMOF-3@Di-2pyk@Mg(II) (**3**).

catalyst	BET specific surface area (m^2^ g^−1^)	total pore volume (cm^3^ g^−1^)	mean pore diameter (nm)	BJH rp, peak (nm)
IRMOF-3	824.6	0.88	2.88	1.22
IRMOF-3@Di-2pyk@Mg(II)	238.9	0.14	2.85	1.22

**Figure 8.**
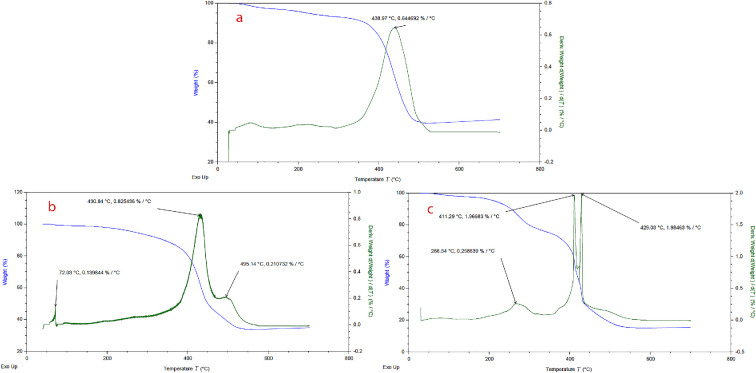
TGA/DTG curves of IRMOF-3 (**1**) (a), IRMOF-3@Di-2pyk (**2**) (b), IRMOF-3@Di-2pyk@Mg(II) (**3**) (c).

TGA was utilized to evaluate the thermal resistance of the ultimate catalyst, IRMOF-3@Di-2pyk@Mg(II) (**3**). The findings are presented in figure 8. In the TGA–DTG profiles, distinct thermal behaviour is observed among the three samples: pristine IRMOF-3, ligand-functionalized IRMOF-3 and the final catalyst **3**. The pristine IRMOF-3 exhibits a major weight loss at approximately 438.97°C with a maximum degradation rate of 0.64% °C^−1^, reflecting high intrinsic thermal stability. Upon functionalization with di(2-pyridyl) ketone, the major decomposition peak slightly shifts to 430.84°C, accompanied by an initial minor weight loss at 72.03°C, attributed to the removal of physically adsorbed organic solvents. The Mg-loaded catalyst displays multiple degradation stages: an initial weight loss at 266.54°C related to embedded organic solvents or coordinated water, and major framework decomposition events at 411.29−429.03°C, with a higher degradation rate (approx. 1.96–1.98% °C^−1^), indicating the structural impact of Mg(II) coordination.

Importantly, despite slight reductions in decomposition temperatures after functionalization and metal loading, the final catalyst retains excellent thermal stability up to around 410°C, which is substantially higher than the operational conditions of the catalytic reactions conducted at room temperature (approx. 25°C). This remarkable thermal robustness confirms that IRMOF-3@Di-2pyk@Mg(II) is highly suitable as a heterogeneous catalyst, providing stability far beyond the reaction environment and ensuring reliable performance under mild aqueous conditions.

## 3. Results and discussion

### 3.1. Catalytic performance of IRMOF-3@Di-2pyk@Mg(II) (3)

The catalytic activity of IRMOF-3@Di-2pyk@Mg(II) was investigated by examining the synthesis of furfuryl alcohol from furfural as a model reaction using 0.002 g of the catalyst. The influence of various factors, such as solvent type, catalyst amount, NaBH_4_ amount, temperature and reaction time, has been systematically investigated (table 2). Among the range of solvents with various polarities (table 2, entries 8−15), H_2_O demonstrated the highest efficiency in the reaction, achieving a 98% yield. Conversely, employing alternative solvents such as THF, dioxane, DCM, and a mixture of ethanol and water (EtOH:H_2_O; 1:1) created a lower yield of furfuryl alcohol.

**Table 2.** Optimization of various factors in the reduction of furfural to furfuryl alcohol catalysed by IRMOF-3@Di-2pyk@Mg(II) (**3**).
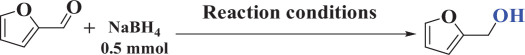

entry	solvent	catalyst amount (mg)	time (min)	temp. (°C)	NaBH_4_ (mmol)	yield (%)^a^
1	H_2_O	2	20	50	0.5	40
2	H_2_O	2	20	70	0.5	48
3	H_2_O	2	40	70	0.5	50
4	H_2_O	2	20	25	0.5	98
5	H_2_O	4	20	25	0.5	98
6	H_2_O	1.5	20	25	0.5	57
7	H_2_O	3	20	25	0.5	98
8	H_2_O	1	20	25	0.5	52
9	EtOH	2	20	25	0.5	61
10	dioxane	2	20	25	0.5	57
11	THF	2	20	25	0.5	35
12	chloroform	2	20	25	0.5	38
13	DMF	2	20	25	0.5	25
14	CH_2_Cl_2_	2	20	25	0.5	20
15	H_2_O/EtOH	2	20	25	0.5	68
16	H_2_O	2	20	25	0.3	95
17	H_2_O	2	20	25	0.25	93
18	H_2_O	catalyst-free	40	25	0.5	10

^a^ Isolated yield

The influence of catalyst quantity was assessed on the reaction yield. The results indicated that increasing the amount of catalyst did not lead to a significant variation in the reaction yield (table 2, entry 5), and less loading of the catalyst ended in a lower yield (table 2, entries 6 and 8). The significant reduction in reaction efficiency to 10% under catalyst-free conditions confirmed the necessity of the catalyst in the reaction environment (table 2, entry 16). Thus, the optimal result was achieved utilizing 0.002 g of the catalyst, 25°C temperature, and H_2_O serving as solvent for the current reaction (table 2, entry 4).

To evaluate the impact of NaBH₄ loading, various amounts were applied in the model reaction (table 2, entry 4). The amount of 0.5 mmol was identified as the optimal quantity, leading to the highest reaction efficiency. Although decreasing the amount of sodium borohydride still afforded acceptable yields (table 2, entries 16 and 17), the efficiencies observed were slightly lower compared to the optimized conditions.

For checking the versatility and capacity of the catalytic reduction of aldehydes and ketones by the present nanocatalyst, a variety of aliphatic and aromatic aldehydes and ketones including biomass-based ones was selected, and their reactivity was considered (table 3). On the basis of these registered data, benzaldehyde and the benzaldehyde derivatives with electron-donating and electron-withdrawing substitutions underwent the catalysed reduction reaction under optimal conditions and afforded the desired benzyl alcohols in excellent yields (table 3, entries 1−9) in the appropriate time, 10 to 20 min. Aliphatic aldehydes were also reduced to the related alcohols in water in less than 25 min with superb yields (table 3, entries 16−18). It is noteworthy that the important biobased alcohols of furfuryl alcohol, cinnamyl alcohol, vanillyl alcohol and 4-hydroxyvaleric acid and citral were also afforded at ambient temperature in water in excellent yields and short reaction times (table 3, entries 9, 12, 15, 17 and 29). Other heteroaromatic aldehydes, such as thiophene-2-carboxaldehyde and pyridine-3-carboxaldehyde, and polynuclear aromatic aldehydes underwent the reduction reaction efficiently and yielded the corresponding primary alcohols (table 3, entries 10, 11, 13 and 14).

The reductions of ketones, despite their steric hindrance, less reactivity, and poor water solubility in comparison to the counterpart aldehydes [54,55], proceeded efficiently (table 3, entries 19−28). These reactions afforded the secondary alcohols in excellent yields at ambient temperature in water and in comparable reaction times. The successful selective reduction of carbonyl compounds, aldehydes and ketones containing nitro (table 3, entries 4 and 8), ester (entry 28) and double bond (entries 15, 17 and 26) functional groups indicates valuable chemoselectivity of IRMOF-3@Di-2pyk@Mg(II).

**Table 3.** The synthesis of primary and secondary alcohols catalysed by IRMOF-3@Di-2pyk@Mg(II) (**3**).
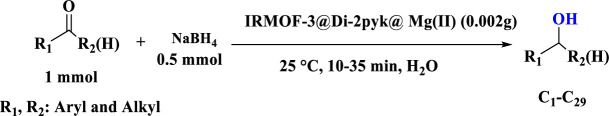

entry	substrate	product
1	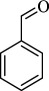	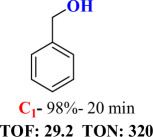
2	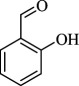	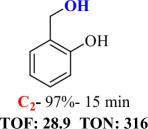
3	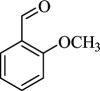	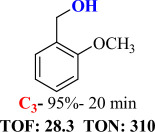
4	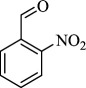	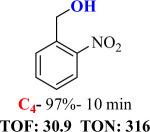
5	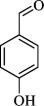	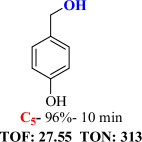
6	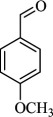	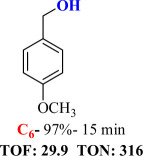
7	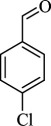	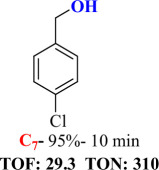
8	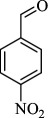	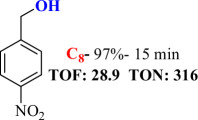
9	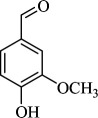	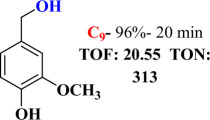
10	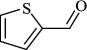	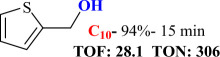
11	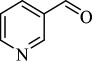	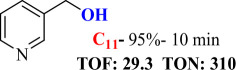
12	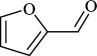	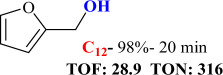
13	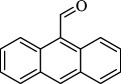	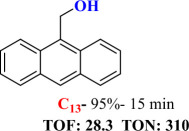
14	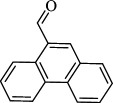	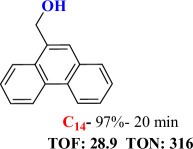
15	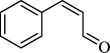	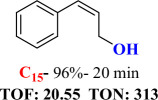
16	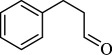	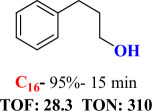
17	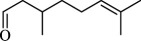	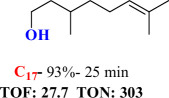
18	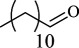	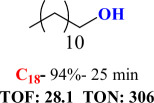
19	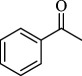	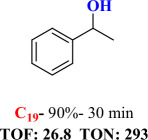
20	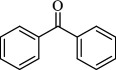	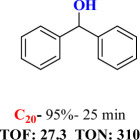
21	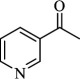	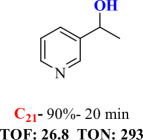
22	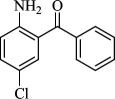	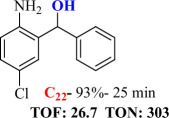
23	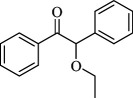	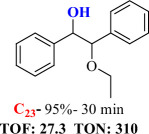
24	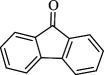	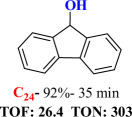
25	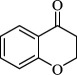	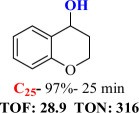
26	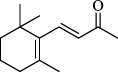	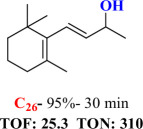
27	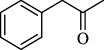	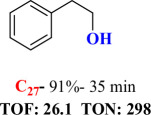
28	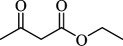	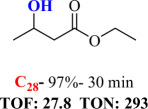
29	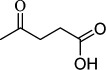	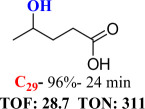

The synergistic effect of Mg(NO_3_)_2_, Di-2pyk, and IRMOF-3, as precursors of IRMOF-3@Di-2pyk@Mg(II) (**3**), on its catalytic activity was investigated in the model reaction, the reduction of benzaldehyde, revealed that **3** exhibits much more catalytic efficiency than its components IRMOF-3 (**1**), Mg(NO_3_)_2_ and IRMOF-3@Di-2pyk (**2**) separately (table 4, entries 1−3).

**Table 4.** Synergistic effects of the different parts of the catalyst.
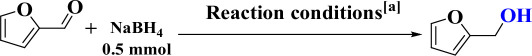

entry	catalyst	time (min)	yield (%)^b^
1	IRMOF-3@Di-2pyk@Mg(II)	20	98
2	IRMOF-3@Di-2pyk	20	27
3	IRMOF-3	20	17
4	Mg(NO_3_)_2_.6H_2_O	20	10

^a^ Reaction conditions: furfural (1 mmol), NaBH_4_ (0.5 mmol), catalyst (0.002 g), H_2_O (2 ml), 25°C. ^b^ Isolated yield.

**Scheme 2.**
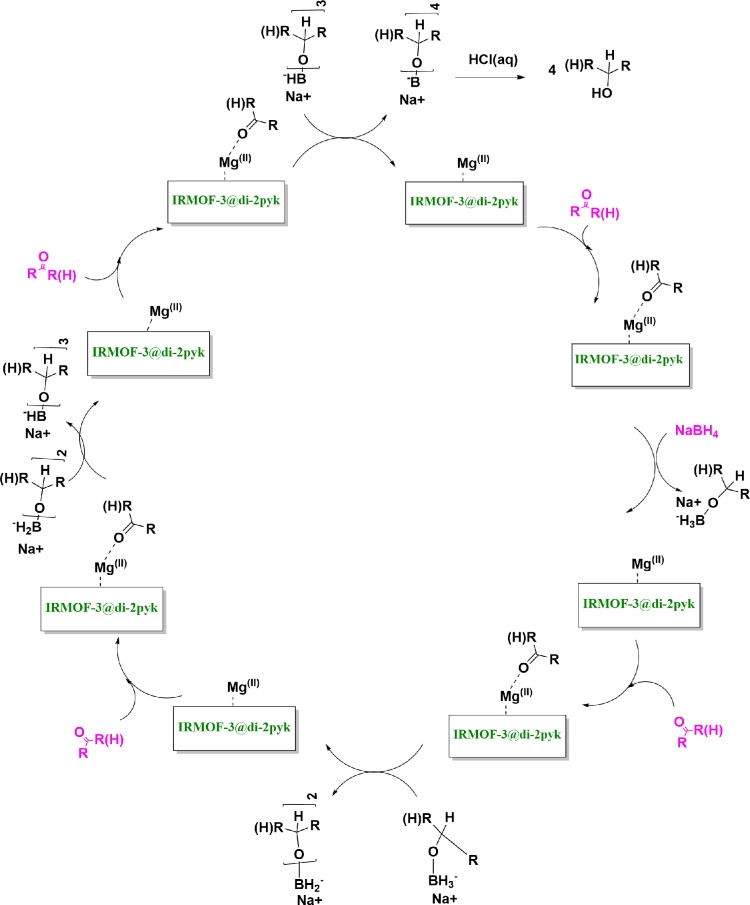
The proposed mechanism for preparation of primary and secondary alcohols.

Our finding about the detailed mechanism for the synthesis of desired primary and secondary alcohols includes carbonyl group activation, hydrogen transfer and acidic workup (Scheme 2) [39,63]. The supported magnesium salt and zinc nodes of IRMOF-3 may activate the carbonyl group and consequently make hydrogen transfer easy, respectively. Moreover, the supported Di-2pyk likely facilitates the interaction between the precursor and the catalyst surface through providing the accessible regions surrounding the catalyst surface. By this process, the reduced product, initially in its alkoxide form, is released from the metal centre. Subsequent protonation yields the alcohol product, thereby regenerating the active catalyst centre.

### 3.2. Recycling and stability of IRMOF-3@Di-2pyk@Mg (II) (3)

**Figure 9.**
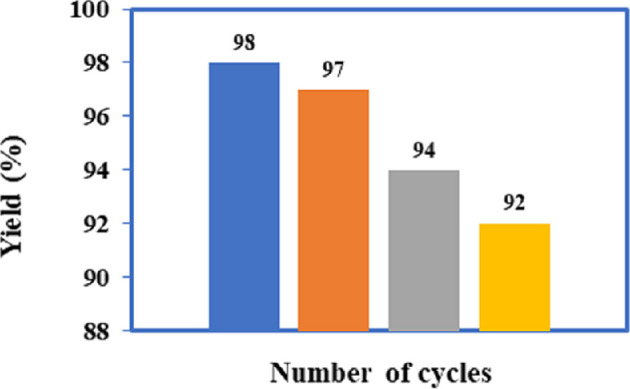
The results of experiments conducted on recycling.

The recyclability and reusability of catalysts are critical factors in their scientific and industrial applications, particularly for large-scale commercial capabilities. Therefore, a recycling experiment was conducted to evaluate the reusability capacity of the modified IRMOF-3 catalyst. The yields of the model reaction exhibited negligible difference after four cycles when catalysed by the recovered IRMOF-3@Di-2pyk@Mg(II), as illustrated in figure 9. In each run, IRMOF-3@Di-2pyk@Mg(II) was separated from the reaction medium by centrifugation, followed by washing with chloroform and ethanol, dried at 80°C under vacuum conditions, and employed in the next experiment. The structure of the catalyst remained intact at the end of the fourth run without any notable change, as verified by XRD, ATR-IR spectroscopy, FESEM (electronic supplementary material, figure S1), and inductively coupled plasma optical emission spectroscopy (ICP-OES) analyses. The occupation of porous space of the MOF due to reactants, products, and solvent absorption [64], and the decrease in magnesium content after the fourth reuse cycle from 4.97 wt% in fresh catalyst to 4.41 wt% measured by ICP-OES suggest a slight deactivation mechanism of the IRMOF-3@Di-2pyk@Mg(II) catalyst.

To assess the chemical stability of catalyst **3**, a hot filtration test [65] was conducted using the documented procedure in the literature [66]. In this procedure, the catalyst was extracted at three separate stages, specifically at 5, 10 and 15 min after the initiation of the reaction. The corresponding yields were determined to be 18, 43 and 77%, respectively. Following the separation of the catalyst, the reaction was allowed to continue for an additional hour. During this time, no significant increase in yield was observed. This observation proves that the IRMOF-3@Di-2pyk@Mg(II) catalyst maintains stability throughout the reaction (electronic supplementary material, figure S2).

Additionally, the catalyst’s efficiency in comparison with that of several previously reported catalysts for synthesizing furfuryl alcohol was investigated (table 5). It was discovered that **3**, as a green and efficient catalyst, demonstrates superiority in point of view of reaction time- and temperature-comparable catalytic effectiveness.

**Table 5.** The comparison of IRMOF-3@Di-2pyk@Mg(II) (**3**) efficiency with that of several other reported catalysts for the preparation of furfuryl alcohol.

entry	catalyst	conditions	time	yield (%)
1	[Ir(COD)Cl]_2_ (0.035 g)	THF/H_2_O, 80°C/NaOH	18 h	99 [67]
2	adZr(IV)/SBA 15 catalysts (0.05 g)	isopropanol/N_2_, 85°C	8 h	95.1 [68]
3	LaFeO_3_ (0.05 g)	EtOH/H_2_/140°C	24 h	70.89 (conv.) [69]
4	Pd/TiH_2_ (0.2)/Pd (0.1 g)	isopropanol/60°C	6 h	18.9 (conv.) [70]
5	MOF808(Hf) (3 mol%)	isopropanol/100°C	1 h	74.6 [63]
6	Ni-SAs/NC (0.02 g)	isopropanol/130°C	3 h	85.1 (conv.) [71]
7	Na− Cu@TS-1 (0.3 g)	isopropanol/H_2_/110°C	2 h	93 (conv.) [72]
8	IRMOF-3@Di-2pyk@Mg(II) (0.002 g)	NaBH_4_, H_2_O, r.t.	20 min	98 (this work)

### 3.3. Gram-scale experiment

To demonstrate the practical applicability of the developed catalyst, a gram-scale reduction of furfural (10 mmol) was carried out using 20 mg of IRMOF-3@Di-2pyk@Mg(II)) and NaBH_4_ in water at room temperature. The reaction was completed within 20 min and afforded furfuryl alcohol in 94% isolated yield. The catalyst was easily recovered by centrifugation, with a negligible mass loss (0.002 g). It was reused for three subsequent runs without significant decrease in yield. These results validate the robustness, efficiency and scalability of the catalytic system under green and mild conditions.

## 4. Conclusion

In the present study, the IRMOF-3@Di-2pyk@Mg(II) nanocatalyst (**3**) was synthesized as a novel, durable and green nanocatalyst and characterized through a number of techniques, including ATR-IR, PXRD, FESEM, EDS for elemental mapping, TEM, BET surface area analysis and TGA.

This nanocatalyst was utilized as a heterogeneous nanocatalyst for the excellent chemoselective and efficient hydrogenation of all types of aldehydes and ketones including biomass-based ones with the addition of sodium borohydride as mild and inexpensive hydrogen source in the presence of other reducible functional groups including nitro, double bond and ester groups under mild and green reaction media at room temperature in short periods of time. The reusability of the catalyst was evaluated through four cycles, revealing consistently high catalytic activity, accompanied by minimal leaching observed from the catalyst upon reuse. The results display that IRMOF-3@Di-2pyk (**2**) serves as an effective support for magnesium nitrate, which also can probably serve for making complexes of other metal salts for application in organic chemistry syntheses.

## Data Availability

The data supporting the findings of this study are available within the article and its electronic supplementary material [73].
